# Eighteen Years of Human Rhinovirus Surveillance in the Republic of Korea (2007–2024): Age- and Season-Specific Trends from a Single-Center Study with Public Health Implications

**DOI:** 10.3390/pathogens14111098

**Published:** 2025-10-28

**Authors:** Yu Jeong Kim, Jeong Su Han, Sung Hun Jang, Jae-Sik Jeon, Jae Kyung Kim

**Affiliations:** 1 Department of Biomedical Laboratory Science, College of Health Sciences, Dankook University, Cheonan-si 31116, Republic of Korea; bkh04117@naver.com (Y.J.K.); jshan1162@naver.com (J.S.H.); zenty87@naver.com (J.-S.J.); 2 Department of Medical Laser, Graduate School of Medicine, Dankook University, Cheonan-si 31116, Republic of Korea; well8143@naver.com; 3 Research Center for Bio-Functional and Biocompatible Materials, Dankook University, Cheonan-si 31116, Republic of Korea

**Keywords:** age distribution, COVID-19 pandemic impact, climate-responsive disease control, epidemiology, molecular surveillance, public health, rhinovirus, seasonality

## Abstract

Human rhinovirus (HRV) is the most common cause of upper respiratory tract infections and can cause substantial morbidity in children. Because its clinical features are nonspecific, differentiation from influenza virus and respiratory syncytial virus is often difficult, underscoring the diagnostic importance of real-time reverse transcriptase polymerase chain reaction (Real-Time RT-PCR)-based detection. This study aimed to characterize long-term epidemiological patterns of HRV in the Republic of Korea and assess their clinical and public health implications. We retrospectively analyzed 23,284 nasopharyngeal swab specimens collected between 2007 and 2024 from outpatients and inpatients presenting with influenza-like illness at a tertiary care hospital. HRV RNA was detected by Real-Time RT-PCR, and positivity rates were compared by year, month, and age group. Annual detection peaked in 2015 (31.3%) and 2016 (28.6%), then dropped sharply during the COVID-19 pandemic (2020–2021, 4.2–11.0%) and remained low through 2024. Seasonally, rates were highest in July (24.4%) and September (24.1%) and lowest in January (6.9%). Age-specific analysis showed peak positivity in children (26.1%) and infants (20.3%), with lower rates in adults (3.9%) and older adults (3.3%). These findings underscore the diagnostic value of HRV detection and provide evidence for pediatric-focused prevention, outbreak preparedness, and climate-informed surveillance strategies.

## 1. Introduction

Respiratory viruses are a major cause of both upper and lower respiratory tract infections globally, with a variety of pathogens responsible for these illnesses [1]. Among them, human rhinovirus (HRV) accounts for over 50% of upper respiratory tract infections and is considered a significant public health concern due to its potential to cause lower respiratory tract infections, particularly in children and immunocompromised individuals [2,3].

HRV was selected as the focus of this study for several reasons. First, its spectrum of clinical manifestations ranges from mild common cold symptoms to severe lower respiratory tract disease, thereby complicating treatment strategies [2,4]. Second, infection patterns vary by age group, children aged 1–12 years showed higher infection rates, whereas adults aged ≥65 years exhibited the lowest infection rates but remained at increased risk of severe outcomes due to comorbidities and immunosenescence [5,6]. Third, HRV exhibits distinct seasonal patterns that are closely associated with climatic and environmental factors, making their analysis particularly valuable [1].

The three HRV species—HRV-A, HRV-B, and HRV-C—differ in terms of infection timing, transmissibility, and clinical presentation. Notably, HRV-A and HRV-C have been associated with more severe disease manifestations [4,7]. However, in the present study, all HRV cases were analyzed collectively without distinguishing between species, which limits the ability to capture species-specific epidemiological and clinical characteristics [2]. Future studies incorporating species-level differentiation will be essential for developing targeted strategies to protect high-risk populations [3,8].

In this study, we aimed to analyze 18 years of HRV testing data from a single tertiary medical center in the Republic of Korea to address a critical gap in the national surveillance system [6,9]. Beyond describing annual, seasonal, and age-specific infection patterns, this study emphasizes the diagnostic implications of HRV detection, particularly its value in differentiating HRV from other respiratory pathogens presenting with influenza-like symptoms [6,10]. By utilizing real-time reverse transcriptase polymerase chain reaction (real-time RT-PCR)-based diagnostics, this study further demonstrates the indispensable role of molecular testing in public health surveillance, enabling the early recognition of epidemic shifts, efficient allocation of healthcare resources, and the development of climate- and policy-informed strategies in the post-pandemic era [9,10].

Moreover, the age-specific infection burden identified in this study underscores the need to strengthen surveillance systems tailored to specific age groups—especially children—to inform more effective preventive measures and targeted intervention strategies [5,6].

## 2. Materials and Methods

### 2.1. Data Collection

This study was approved by the Institutional Review Board of Dankook University, Cheonan, the Republic of Korea (approval number: DKU 2025-02-004-003), and conducted in accordance with the ethical principles of the Declaration of Helsinki. As only fully anonymized retrospective data were used, the requirement for informed consent was waived.

HRV testing data were collected from a single tertiary medical center, Dankook University Hospital, between 2007 and 2024. The dataset included test date, test result, and patient age, with a total of 23,284 specimens analyzed. Testing was primarily performed in outpatients and inpatients presenting with influenza-like symptoms such as fever, cough, or sore throat, or for the differential diagnosis of respiratory infections. Specimens lacking essential demographic information were excluded from the analysis. As the dataset reflects hospital-based diagnostic testing requested by attending clinicians, it differs from community-based sentinel surveillance in target population, testing indications, and sampling intensity.

### 2.2. Specimen Collection and Data Analysis

All nasopharyngeal swab specimens were collected by clinical physicians using gamma-sterilized flocked swabs, following the Centers for Disease Control and Prevention (CDC) clinical specimen collection and handling guidelines [11]. During collection, the patient’s head was slightly tilted (approximately 70°), and the swab was gently inserted parallel to the nasal floor until mild resistance was encountered at the posterior nasopharynx. The swab was rotated several times and withdrawn slowly. Each specimen was then placed in a sterile transport tube, sealed, and stored in a dedicated 4 °C specimen refrigerator. Samples were tested immediately whenever possible; if immediate processing was not feasible, all specimens were analyzed within 24 h of collection.

In accordance with the International Council for Harmonisation E11 guidelines, study subjects were categorized into five age groups: infants (<1 year), children (1–12 years), adolescents (13–18 years), adults (19–64 years), and older adults (≥65 years). This classification, commonly applied in clinical and epidemiological research, was consistently used throughout the study. Seasons were defined by calendar month at the time of testing, and year-to-year trends were analyzed to compare pre- and post-pandemic periods.

### 2.3. RNA Extraction and Real-Time Reverse Transcriptase Polymerase Chain Reaction (Real-Time RT-PCR)

Nasopharyngeal swab specimens were processed immediately after collection or stored at 4 °C and tested within 24 h. Viral RNA was extracted using the QIAamp Viral RNA Mini Kit (Qiagen, Hilden, Germany) and eluted with RNase-free water according to the manufacturer’s instructions.

Extracted RNA was analyzed by real-time RT-PCR using the AdvanSure™ RV Plus RT-PCR Kit (LG Life Sciences, Seoul, Republic of Korea), a commercially validated multiplex assay for the simultaneous detection of 15 respiratory viruses, including human rhinovirus. Amplification and fluorescence detection were performed on the SLAN Real-Time RT-PCR System (LG Life Sciences, Seoul, Republic of Korea), following the manufacturer’s protocol. The analytical performance of this assay has previously demonstrated a sensitivity of ≤10^2^ copies/reaction and specificity of ≥98% [12].

### 2.4. Statistical Analysis

All statistical analyses were performed using R software (version 4.5.1). The primary outcome variable was HRV positivity, while most independent variables (e.g., age group, season) were categorical. For comparisons across two or more groups (e.g., seasonal or age-specific HRV positivity rates), associations between variables were evaluated by two-tailed chi-square tests to compare expected and observed distributions under the null hypothesis. A *p*-value < 0.05 was considered statistically significant.

## 3. Results

### 3.1. Baseline Characteristics of the Study Population

A total of 23,284 respiratory specimens were tested for HRV during the study period, comprising 13,961 (60.0%) males and 9323 (40.0%) females. The median age of the tested population was 3 years (interquartile range = 0.8–55 years), indicating that young children accounted for the majority of the study cohort. The overall age distribution showed that the largest proportion of samples originated from children aged 1–12 years (45.3%), followed by older adults aged ≥65 years (20.2%) and infants <1 year (19.5%). Adults (19–64 years) and adolescents (13–18 years) comprised 12.6% and 2.5% of all tested individuals, respectively (Table 1).

### 3.2. Annual HRV Positivity Trend (2007–2024)

Analysis of the annual number of HRV-positive cases revealed significant fluctuations over the 18-year study period (χ^2^ = 905.52, df = 17, *p* < 0.001). From 2007 to 2009, the number of positive cases remained relatively stable at approximately 140–180. In 2010, the number increased to 335 cases, followed by fluctuations between 246 and 320 cases from 2011 to 2014.

In 2015 (435 cases) and 2016 (472 cases), HRV detections reached the highest levels observed during the study period. Thereafter, a gradual decline was recorded, with 416 cases in 2017, 287 in 2018, and 211 in 2019. Beginning in 2020, the number of positive cases dropped sharply to just 34, and from 2021 to 2024, fewer than 75 cases were reported annually (Table S1).

**Figure 1:**
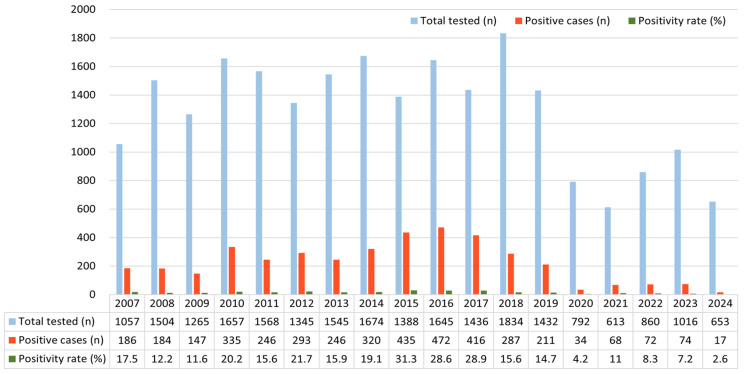
Annual number of total tested cases, HRV-positive cases, and corresponding positivity rates (%) from 2007 to 2024. Light blue bars indicate the total number of specimens tested each year, orange bars represent the number of HRV-positive cases, and the green line denotes the annual positivity rate (%). The figure illustrates distinct annual fluctuations in HRV detection, with a marked decline during the COVID-19 pandemic (2020–2021) followed by persistently reduced activity through 2024. Abbreviation: HRV, human rhinovirus.

A modest resurgence was observed in 2022 and 2023, with 72 and 74 cases, respectively, but the numbers remained lower than pre-pandemic levels. In 2024, only 17 cases were identified. However, this decline occurred alongside a marked reduction in specimen numbers, indicating that the observed trend may reflect both reduced viral circulation and decreased number of tests (Figure 1).

### 3.3. Seasonal HRV Positivity Rate

**Figure 2:**
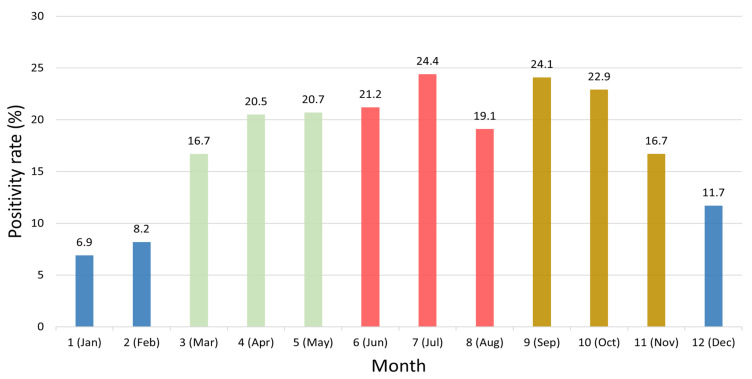
Monthly variation in human rhinovirus (HRV) positivity rates from 2007 to 2024. Bars represent monthly HRV positivity rates (%) calculated from 2007 to 2024. Distinct color tones indicate seasonal groupings: blue for winter (December–February), light green for spring (March–May), red for summer (June–August), and golden brown for autumn (September–November). The figure highlights pronounced HRV activity during the late spring (May) to early autumn (September) months, peaking in July (24.4%).

Monthly analysis of HRV positivity rates revealed significant variation (χ^2^ = 517.41, df = 11, *p* < 0.001; Table 2). The lowest positivity rate was observed in January (6.9%), with similarly low levels in February (8.2%). From March (16.7%) to June (21.2%), a gradual increase was noted. The highest positivity rate was recorded in July (24.4%), while high levels were also observed in September (24.1%) and October (22.9%). In contrast, the rate declined to 16.7% in November and further to 11.7% in December (Figure 2).

### 3.4. HRV Positivity Rate by Age Group

**Figure 3:**
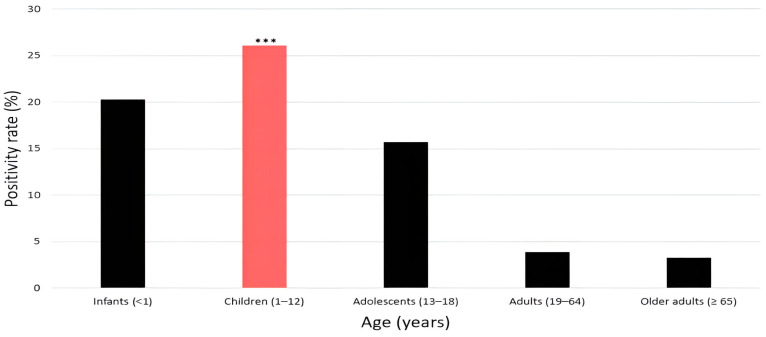
Age-specific positivity rates of human rhinovirus (HRV). Bars represent HRV positivity rates (%) by age group, showing the highest rate in children (red bar) (26.1%), followed by infants (20.3%) and adolescents (15.7%), while adults (3.9%) and older adults (3.3%) exhibited markedly lower rates; significance is indicated as *** *p* < 0.001.

Among the 23,284 individuals tested, HRV positivity rates differed significantly across age groups (χ^2^ = 1596.4, df = 4, *p* < 0.001; Table 3). In infants (<1 year), the positivity rate was 20.3% (924/4543). In children (1–12 years), the rate was 26.1% (2752/10,537), representing the highest among all age groups. Adolescents (13–18 years) showed a positivity rate of 15.7% (91/577). Adults (19–64 years) exhibited a comparatively low positivity rate of 3.9% (117/2935). Older adults (≥65 years) had the lowest rate, 3.3% (159/4692), among all age groups (Figure 3).

## 4. Discussion

This 18-year analysis provides one of the most comprehensive characterizations of HRV epidemiology in Korea, identifying clinically and public health-relevant patterns across annual, seasonal, and age-specific strata [13,14].

While several global studies have reported a marked resurgence of respiratory viruses after the relaxation of COVID-19 restrictions, our single-center data showed a sustained decline in HRV detections. This divergence likely reflects several interacting factors: (i) lasting impact of non-pharmaceutical interventions such as mask wearing, hand hygiene, and social distancing, which reduced actual transmission; (ii) reduced hospital attendance and altered healthcare-seeking behavior, resulting in fewer diagnostic specimens; (iii) a shift toward testing predominantly severe or high-risk cases, thereby influencing positivity estimates; and (iv) delayed recovery of population-level immunity gaps that may have postponed the typical seasonal rebound. Collectively, these factors highlight how post-pandemic epidemiological patterns observed in hospital-based diagnostic data may differ from those captured by community-level surveillance.

Our seasonal trends differed from those reported by South Korea’s nationwide respiratory virus surveillance and from institutional reports [15,16,17]. These discrepancies likely stem from differences in study design, surveillance scope, and population characteristics. National sentinel data are community-based and short-term (mainly 2019–2022), whereas our dataset reflects hospital-based diagnostic testing over 18-years (2007–2024) in a tertiary-care hospital setting. This distinction affects the observed timing and magnitude of seasonal peaks because testing intensity, patient age composition, and healthcare-seeking behavior—especially during 2020–2021 non-pharmaceutical interventions—differ markedly between systems. Although single-center data may not fully represent nationwide trends, the longitudinal design provides unique insight into long-term shifts and post-pandemic recovery dynamics, complementing national surveillance findings rather than contradicting them.

One of the most notable findings was the pronounced HRV positivity observed in July (24.4%) and September (24.1%). This refined summer–early autumn predominance contrasts with the spring and autumn peaks reported in temperate Europe [18], underscoring climatic and behavioral drivers of HRV seasonality in East Asia. Similar summer peaks have also been documented in subtropical and tropical regions [18,19], where hot and humid weather, widespread use of air conditioning, and high population density may contribute to HRV transmission in Korea. Furthermore, comparable summer peaks have been reported in Japan [20]. In Japan, HRV circulation has been reported to occur year-round, although the predominant period of activity varies by region and study period. Recent surveillance data demonstrated notable peaks during summer to autumn [21], whereas a regional study in Nara Prefecture observed a marked rise in HRV detections during summer [22]. These findings indicate that HRV seasonality in Japan may fluctuate across different climatic zones and surveillance settings, indicating that the seasonal distribution observed in Korea reflects a broader East-Asian regional phenomenon rather than a uniquely national trend [14,20]. The absolute difference between mean positivity rates in summer (21.5%) and winter (8.9%) was 12.6 percentage points, exceeding the 5% threshold proposed in methodological literature for clinical relevance [23]. This suggests that the seasonal variation in HRV positivity is not only statistically significant but also clinically meaningful, underscoring the importance of intensified surveillance and preventive measures. Although no HRV vaccine is currently available, these findings may serve as fundamental evidence to inform future vaccine development and public health preparedness prior to the summer and early autumn peak periods [24].

During the COVID-19 pandemic (2020–2021), non-pharmaceutical interventions such as mask-wearing and social distancing helped reduce HRV exposure and affected population-level immunity [25,26]. HRV activity declined markedly and appeared not to return to pre-pandemic levels. However, the number of respiratory specimens also decreased substantially during this period, which may have contributed to the observed reduction. Thus, caution is warranted in attributing the decline solely to epidemiological factors. The concept of ‘immunity debt,’ reflecting increased susceptibility in children after prolonged viral suppression, remains plausible but cannot be confirmed in this dataset [27].

Rhinovirus infections in adults represent an emerging public health concern be yond being a mild upper respiratory illness. Traditionally considered a pediatric pathogen, HRV is increasingly implicated in exacerbations of chronic respiratory and cardiovascular diseases, adult pneumonia, and lower respiratory tract infections, often resulting in prolonged hospitalization and greater healthcare resource use [26]. In our study, RV positivity was detectable in adults (3.9%) and older adults (3.3%), showing that, although lower than in children, RV continuously circulates across all age groups. These results align with recent reports and emphasize the need for ongoing surveillance of adult RV infections and broader recognition of their clinical and epidemiological significance [14].

Age-stratified analysis revealed that children aged 1–12 years had the highest positivity rate (26.1%), reaffirming their role as the most vulnerable group and a major reservoir for HRV transmission [28]. HRV infection in children is frequently associated with hospitalization, bronchiolitis, and wheezing [29], imposing a substantial burden on pediatric clinical practice. A recent Korean multicenter study likewise reported that HRV accounted for a significant proportion of pediatric respiratory hospitalizations [28]. These findings strongly support the need for pediatric-focused vaccination development and infection control strategies [27].

In recently proposed public health frameworks, an absolute difference of ≥5 percentage points in outcome measures is considered clinically meaningful [19]. In this study, the positivity rate among children exceeded that of adults (3.9%) and older adults (3.3%) by more than 22 percentage points, far surpassing this threshold. This highlights the need for age-targeted preventive strategies and reconfirms the disproportionate burden of HRV infection in pediatric populations [29].

This single-center, hospital-based diagnostic study may not fully represent community-level circulation patterns captured by national sentinel surveillance. Differences in sampling frame, testing indications, case mix, and healthcare access—particularly during 2020–2021—likely influenced the observed seasonal timing and magnitude. Therefore, our findings should be interpreted as complementary to, rather than interchangeable with, national surveillance estimates [26]. Broader national surveillance systems, such as Egypt’s nationwide framework for influenza-associated acute respiratory infections [30], provide more comprehensive and representative data for guiding public health policies. Second, the diagnostic assay employed did not distinguish among HRV genotypes. HRV-A, B, and C differ in pathogenicity and seasonal distribution, but the assay used in this study was designed to detect HRV collectively [31]. Nevertheless, aggregate analysis provided valuable insights into the overall HRV burden, and given the association of HRV-C with severe lower respiratory tract infections in children, genotype-specific surveillance is needed [24,28]. HRV-A and HRV-C each exhibit distinct epidemiological and clinical features, such as year-round circulation of HRV-A and the strong association of HRV-C with severe disease [24]. Failure to distinguish between genotypes may, therefore, result in over- or underestimation of their respective contributions. Future multicenter, genotype-based studies are warranted [19,28]. Another limitation is the variation in annual specimen numbers, particularly during the pandemic (2020–2024), when number of specimens dropped sharply, which may have influenced temporal interpretations of HRV positivity trends.

Simulation-based power estimation methods could help clarify whether observed differences reflect true epidemiological changes or sampling variability [32].

Nevertheless, the major strength of this study lies in its large-scale dataset comprising 23,284 respiratory specimens collected over 18 years. The consistent statistical significance observed across seasonal and age-specific comparisons indicates that the study had sufficient power to detect meaningful differences. This reduces the likelihood of Type II error and enhances the reliability of the identified epidemiological trends [33,34]. Future research should incorporate genotype-specific diagnostic methods to achieve a more precise understanding of HRV epidemiology and its clinical burden [35,36].

Moreover, aligning national surveillance data with globally recognized frameworks, such as those established by the WHO, will facilitate cross-country comparisons and support coordinated public health responses [37]. Building upon the present findings, in our future work, we intend to conduct a multicenter study that encompasses several respiratory viruses within an integrated analytical framework. Our previous work analyzed the epidemiological patterns of 15 respiratory viruses, thereby establishing a comparative foundation for subsequent investigations [38]. Based on this evidence, large-scale multicenter and multinational studies are warranted to elucidate inter-virus interactions and concurrent circulation dynamics more comprehensively, advancing a global understanding of respiratory virus epidemiology.

## 5. Conclusions

This 18-year retrospective analysis provides comprehensive insights into the epidemiological trends of human rhinovirus infections in a single tertiary hospital in Korea, highlighting distinct age-specific susceptibility, seasonal variation, and substantial shifts in circulation patterns before and after the COVID-19 pandemic. Our findings indicate that hospital-based diagnostic data, while complementary to national surveillance, reveal unique dynamics influenced by healthcare utilization, public health interventions, and immunity gaps. These results underscore the need for continued monitoring of adult infections and age-stratified strategies to mitigate respiratory virus burden. Future multicenter studies integrating broader population data will be essential to refine surveillance systems and inform targeted prevention policies.

## Figures and Tables

**Table 1:** Distribution of the study participants (*n* = 23,284) by age group, positivity rate (%) and sex.

Age Group	Male (*n*)	Female (*n*)	Total (*n*)	Positivity Rate (%)
Infants (<1 year)	2704	1839	4543 (19.5%)	20.3
Children (1–12 years)	5992	4545	10,537 (45.3%)	26.1
Adolescents (13–18 years)	336	241	577 (2.5%)	15.7
Adults (19–64 years)	1893	1042	2935 (12.6%)	3.9
Older adults (≥65 years)	3036	1656	4692 (20.2%)	3.3
Total	13,961	9323	23,284 (100%)	

**Table 2:** Monthly distribution of human rhinovirus (HRV) positivity rates, 2007–2024.

Month	Total Tested (*n*)	Positive Case (*n*)	Positivity Rate (%)	*p*-Value
1 (January)	2118	148	6.9	<0.001
2 (February)	1813	150	8.2	
3 (March)	1912	321	16.7	
4 (April)	2180	447	20.5	
5 (May)	2299	478	20.7	
6 (June)	1736	369	21.2	
7 (July)	1535	376	24.4	
8 (August)	1539	295	19.1	
9 (September)	1496	361	24.1	
10 (October)	1794	412	22.9	
11 (November)	2317	387	16.7	
12 (December)	2545	299	11.7	

**Table 3:** Age-specific distribution of human rhinovirus (HRV) positivity rates with statistical comparison.

Age Group	Total Tested (*n*)	Positive Case (*n*)	Negative Case (*n*)	Positivity Rate (%)	*p*-Value
Infants (<1 year)	4543	924	3619	20.3	<0.001
Children (1–12 years)	10,537	2752	7785	26.1	
Adolescents (13–18 years)	577	91	486	15.7	
Adults (19–64 years)	2935	117	2818	3.9	
Older adults (≥65 years)	4692	159	4533	3.3	

## Data Availability

The data that support the findings of this study are derived from patient records at Dankook University Hospital and are subject to ethical and legal restrictions. Due to privacy and confidentiality concerns, the raw datasets cannot be made publicly available. However, anonymized summary data may be obtained from the corresponding author upon reasonable request, subject to approval by the Institutional Review Board.

## Supplementary Materials

The following supporting information can be downloaded at: https://www.mdpi.com/article/10.3390/pathogens14111098/s1, Table S1. Annual number of HRV tests, positive cases, and positivity rates (2007–2024). Legend. Annual distribution of human rhinovirus (HRV) testing, positive detections, and positivity rates over 18 years (2007–2024). Peaks were observed in 2015–2016, followed by a sharp decline during the COVID-19 pandemic, and persistently low levels thereafter.

## Abbreviations

The following abbreviations are used in this manuscript:

HRV	Human rhinovirus
real-time RT-PCR	Real-time reverse transcriptase polymerase chain reaction
