# Supplementary material for: Eighteen Years of Human Rhinovirus Surveillance in the Republic of Korea (2007–2024): Age- and Season-Specific Trends from a Single-Center Study with Public Health Implications
Source: Pathogens. 2025 Oct 28;14(11):1098. doi: 10.3390/pathogens14111098 (PMC12655215; doi:10.3390/pathogens14111098)
Supplement: Supplementary file 1 [file pathogens-14-01098-s001.zip › pathogens-3925783-supplementary.pdf]

Table S1. Annual number of HRV tests, positive cases, and positivity rates (2007–2024).

Year	Total tested (n)	Positive cases (n)	Positivity rate (%)
2007	1,057	186	17.5
2008	1,504	184	12.2
2009	1,265	147	11.6
2010	1,657	335	20.2
2011	1,568	246	15.6
2012	1,345	293	21.7
2013	1,545	246	15.9
2014	1,674	320	19.1
2015	1,388	435	31.3
2016	1,645	472	28.6
2017	1,436	416	28.9
2018	1,834	287	15.6
2019	1,432	211	14.7
2020	792	34	4.2
2021	613	68	11.0
2022	860	72	8.3
2023	1,016	74	7.2
2024	653	17	2.6

Annual distribution of human rhinovirus (HRV) testing, positive detections, and positivity rates over 18 years (2007–2024). Peaks were observed in 2015–2016, followed by a sharp decline during the COVID-19 pandemic, and persistently low levels thereafter.
